# Hypoglycemic and hypolipidemic effect of S-allyl-cysteine sulfoxide (alliin) in DIO mice

**DOI:** 10.1038/s41598-018-21421-x

**Published:** 2018-02-23

**Authors:** Baiqiang Zhai, Chuanhai Zhang, Yao Sheng, Changhui Zhao, Xiaoyun He, Wentao Xu, Kunlun Huang, Yunbo Luo

**Affiliations:** 1 Beijing Advanced Innovation Center for Food Nutrition and Human Health, College of Food Science and Nutritional Engineering, China Agricultural University, Beijing, 100083 China; 2 Key Laboratory of Safety Assessment of Genetically Modified Organism (Food Safety), Ministry of Agriculture, Beijing, 100083 China; 3 Department of Food Quality and Safety, College of Food Science and Engineering, Jilin University, Changchun, Jilin Province 130062 China

## Abstract

Alliin (S-allyl cysteine sulfoxide) is a bioactive sulfoxide compound derived from garlic. To evaluate the preventive effect of alliin against metabolic risk factors in diet induced obese (DIO) mice, we treated the C57BL/6J DIO mice with drinking water with or without alliin (0.1 mg/ml) for 8 weeks. Results showed that alliin had no significant effect on the body weight, adiposity or energy balance. However, alliin treatment enhanced glucose homeostasis, increased insulin sensitivity and improved the lipid profile in the DIO mice. This was, at least partly, attributable to alliin induced modulation of the intestinal microbiota composition, typically decreased *Lachnospiraceae* and increased *Ruminococcaceae*. From above, we conclude that alliin has nutraceutical or even medicinal potential in prevention of diabetes and lipid metabolic disorders.

## Introduction

Garlic (*Allium sativum L*.) is not only a traditional flavoring agent but also possesses nutraceutical or therapeutic effect for many diseases including atherosclerosis, cardiovascular diseases, hyperlipidemia, hypertension and various cancers^1–4^. The main identified active ingredient in garlic is alliin (S-allyl cysteine sulfoxide) - a natural sulfoxide derived from the amino acid cysteine^3,5^, which accounts for 6 to 14 mg of alliin per gram of fresh garlic^6^ and accumulates naturally during the storage of garlic bulbs at cool temperature^3^. When garlic tissue is damaged, the alliinase released from cell vacuoles immediately converts alliin into allicin, also called garlicin^7^.

Allicin is highly unstable and nearly none in most commercial garlic products^3^. On the other hand, water-soluble organosulfur compounds derived from garlic such as alliin are stable, odorless and safe^8^, which also constitutes fresh garlic or garlic powder^3^. Different from allicin that is quickly metabolized into diallyl disulfide and allyl mercaptan, alliin with bioavailability of 16.5% has nearly no first liver pass effect except that some is converted to diallyl disulfide^8,9^. Extensive literature review discloses the nutraceutical and medicinal potential of alliin in several aspects. For example, alliin decreased fasting glucose level and increased insulin level to the same extent as did glibenclamide, glyclazide or insulin in diabetic rats^10,11^. Weeks’ administration of alliin modified the redox environment by decreasing reactive substances in organs and increasing the activities of catalase and superoxide dismutase in nicotine fed rats^12^. Another study showed that alliin markedly depressed the increase of plasma and liver cholesterol level in rats fed a hypercholesterolemic diet containing 10% hydrogenated coconut oil, 1% cholesterol and 0.2% cholic acid^13^. Alliin was also found to possess anti-inflammatory activities for bowel diseases^14^. These symptoms like diabetes, high radicals, hypercholesterolemia and inflammation are all associated with obesity and/or metabolic diseases. Supportively, a meta-analysis suggests that the single component intake of garlic may be effective for lowering plasma glucose concentration and body weight^15^.

Metabolic diseases are spreading so quickly like a plague worldwide that the research to curb this trend is urgent. Exploring the bioactive compounds from kingdom of natural plants that can prevent the metabolic risk factors has been becoming a popular strategy and thus has caught much attention^16–18^. Therefore, we intended to systematically evaluate the preventive effect of alliin against metabolic risk factors in diet induced obese (DIO) mice.

## Results

### Alliin maintains glucose homeostasis and improves insulin sensitivity

**Figure 1:**
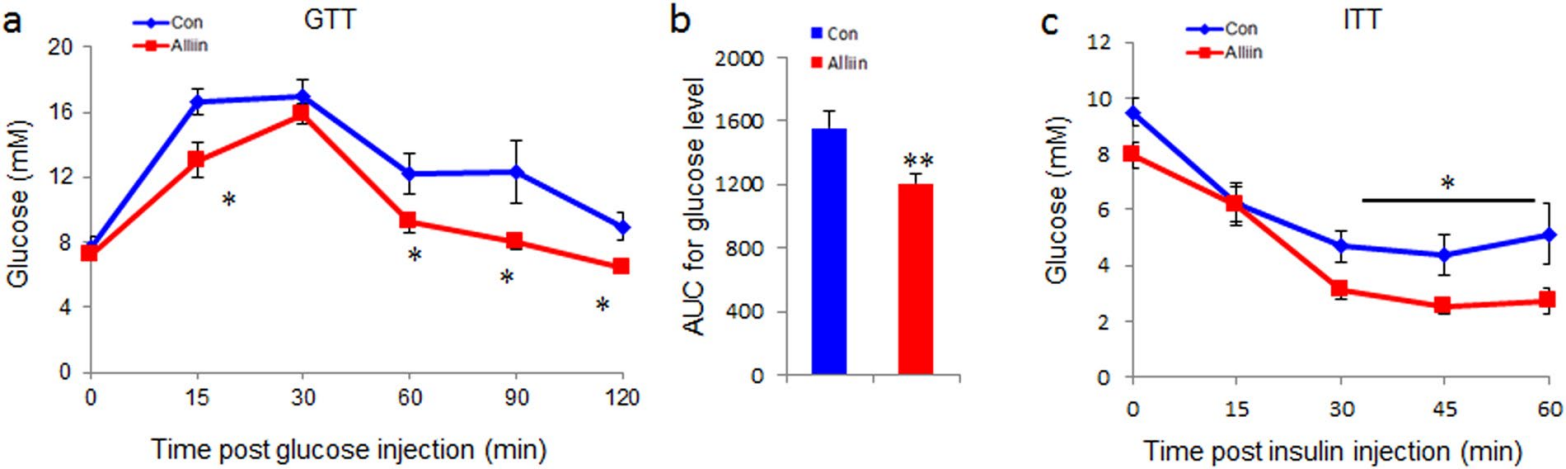
Alliin improves glucose tolerance and insulin sensitivity in DIO mice. (**a** and **b**) Alliin significantly reduced glucose level challenged with insulin in DIO mice. (**c**) Alliin significantly improved insulin sensitivity to glucose injection in DIO mice. Data are means ± SEM (n = 10). *P < 0.05, **P < 0.01. GTT, glucose tolerance test; ITT, insulin tolerance test.

Alliin effectively reduced fasting glucose level in the blood. Specifically, alliin treated mice had lower fasting glucose level post injection of insulin at 15, 60, 90 and 120 min in DIO mice (P < 0.05) (Fig. 1a,b). Alliin also significantly increased insulin sensitivity to glucose injection in DIO mice. Glucose level in alliin treated mice was significantly lower than that of the control mice at 30, 45 and 60 min post injection of glucose (Fig. 1c).

### Alliin improves lipid profile in DIO mice

**Figure 2:**
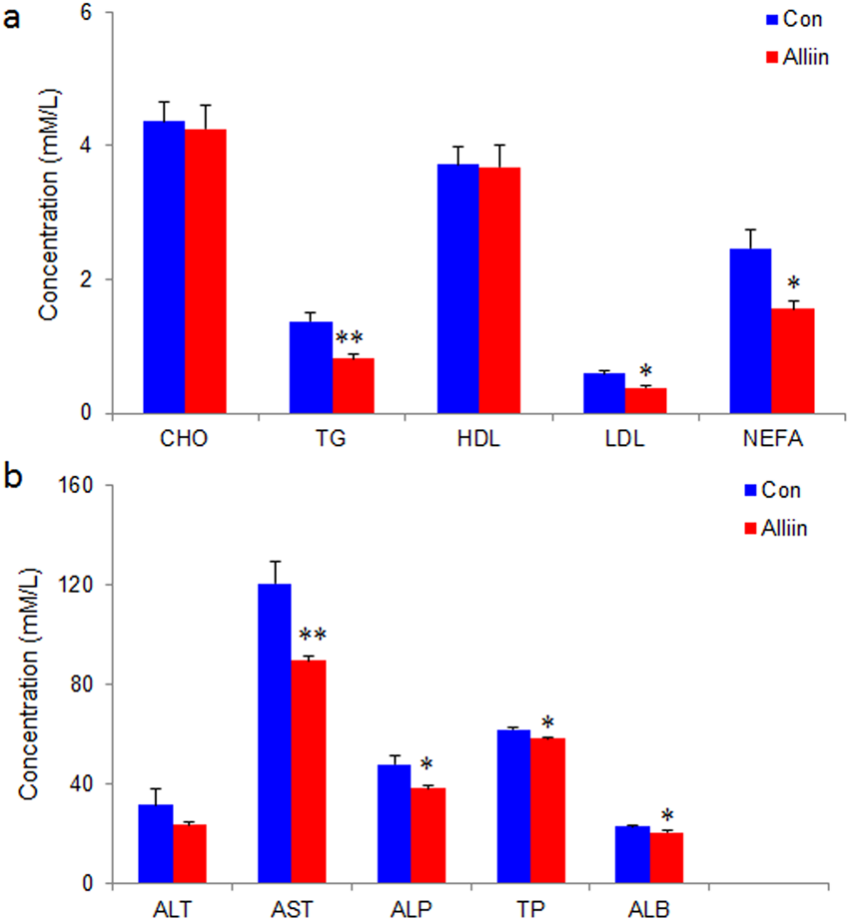
Biochemistry of blood of alliin treated DIO mice. Alliin improved (**a**) the lipid profile and (**b**) the liver function of DIO mice. Data are means ± SEM (n = 10). *P < 0.05, **P < 0.01. CHO, cholesterol; TG, triglyceride; HDL, high density lipoprotein; LDL, low density lipoprotein; NEFA, non-esterized free fatty acid; ALT, alanine aminotransferase; AST, aspartate aminotransferase; ALP:Palkaline phosphatase; TP, total protein; ALB, albumin.

Alliin decreased both total triglycerides (TG) and free fatty acids (FFA) in DIO mice while increased high density lipoprotein (HDL). No significant difference was found in total cholesterol (TC) and low density lipoprotein (LDL) (Fig. 2a). Alliin also showed protective effect on liver of DIO mice by decreasing several indices like aspartate aminotransferase (AST), alkaline phosphatase (ALP), total protein (TP) and albumin (ALB) (Fig. 2b).

### Alliin and adiposity of DIO mice

**Figure 3:**
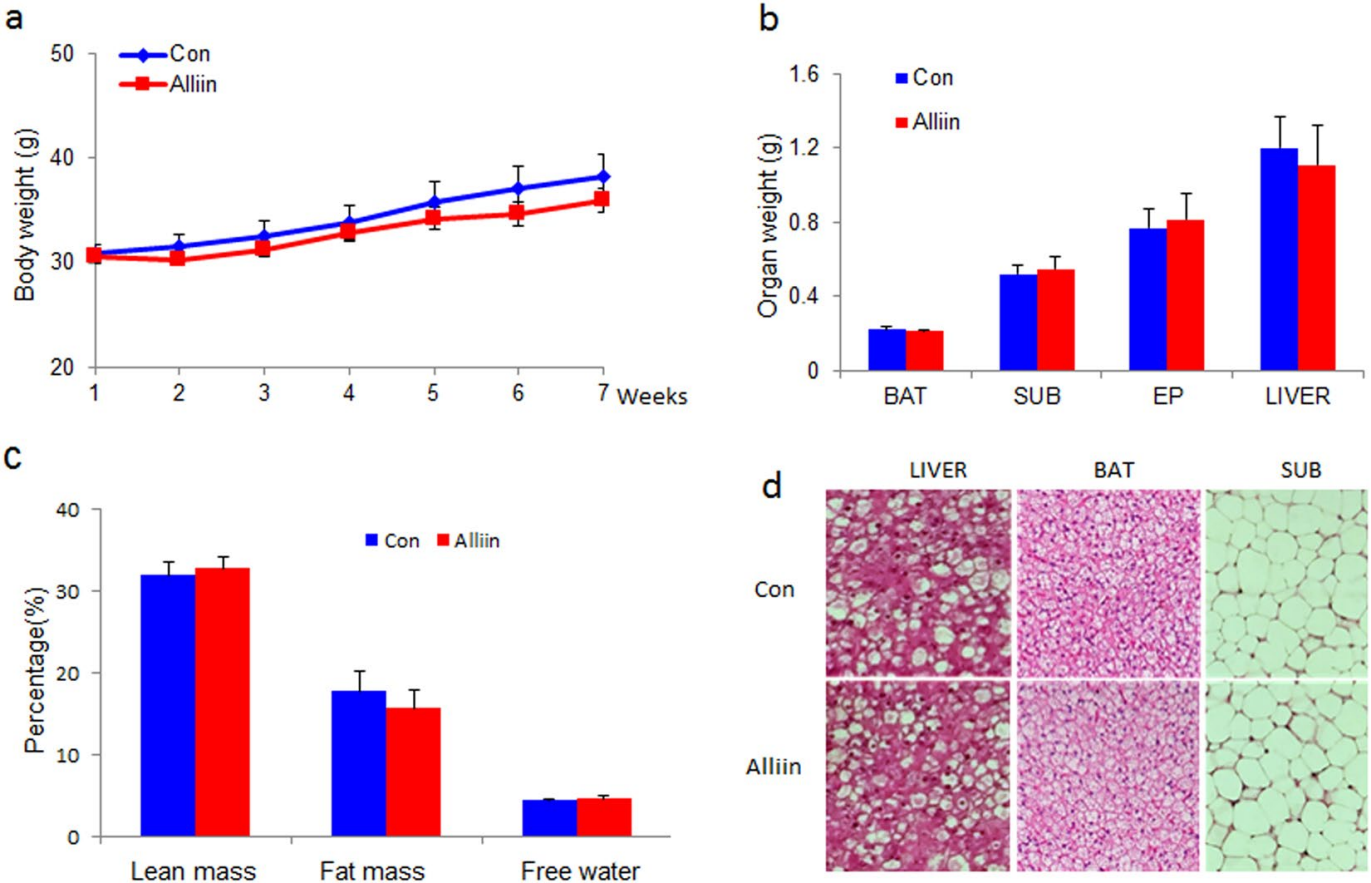
Alliin has no significant effect on body weight, organ weight or fat mass in DIO mass. (**a**,**b** and **c**) No significant difference of body weight, organ weight or fat content was found between alliin treated or control mice. (**d**) HE staining showed a similar tissue structure of liver, BAT and subcutaneous fat mass between alliin treated and the control mice. Representative pictures were shown. Data are means ± SEM (n = 6). BAT, brown adipose tissue; SUB, subcutaneous fat. BAT, brown adipose tissue; SUB, subcutaneous fat; EP, epididymal fat.

Over one-month administration of alliin failed to prevent the body weight gain of the mice fed with high fat (Fig. 3a). No significant difference was found in the weight of liver and fat tissues (brown adipose tissue, BAT; epididymal fat, EP; subcutaneous fat, Sub), the body composition (lean mass, fat mass and free water) or the histological structure (Fig. 3b,c).

### Alliin and energy balance of DIO mice

**Figure 4:**
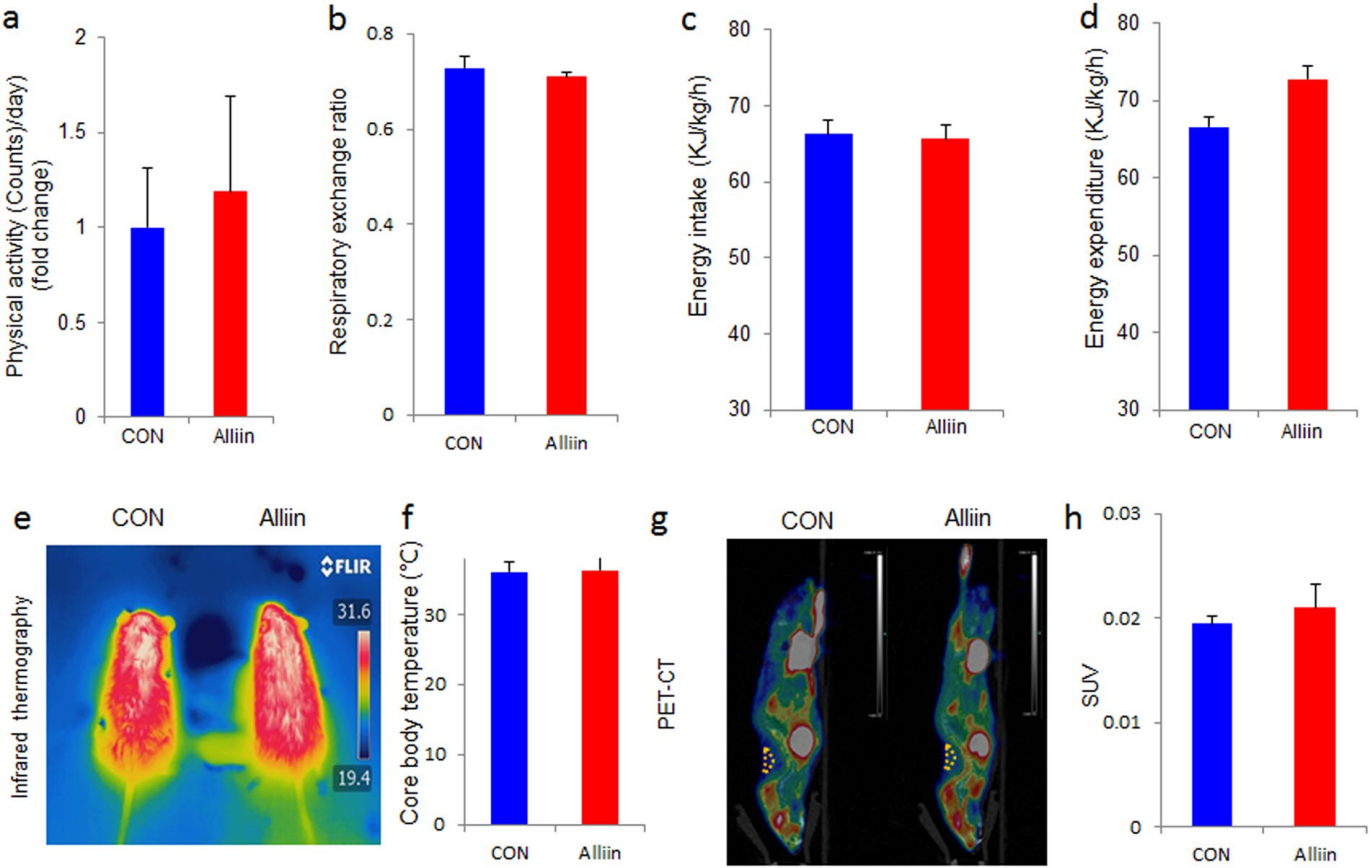
Alliin does not change the whole-body energy metabolism in DIO mice. Alliin did not affect (**a**) the physical activity, (**b**) the respiratory exchange ratio, (**c**) energy intake or (**d**) energy expenditure. No significant difference of (**e** and **f**) the core body temperature between alliin treated and the control DIO mice under cold condition. Both (**g**) the sagittal view of PET-CT images and (**h**) quantified SUV showed that alliin treatment did not affect brown adipose tissue activity in DIO mice. Data are means ± SEM (n = 6). Yellow triangle: anatomic site of interscapular BAT. SUV, standard uptake value.

The alliin treated mice consumed 4.35 ± 0.44 ml water (equivalent to approximately 0.44 mg alliin) the day before the whole-body energy metabolism of the DIO mice was examined. Results showed that the physical activity, respiratory exchange rate, energy intake or total energy expenditure was not changed by alliin treatment in DIO mice (Fig. 4a–c). Similarly, alliin did not change the thermography, the body core temperature or the associated activity of brown adipose tissue in DIO mice (Fig. 4d–g).

### Alliin modifies intestinal microbiota of DIO mice

**Figure 5:**
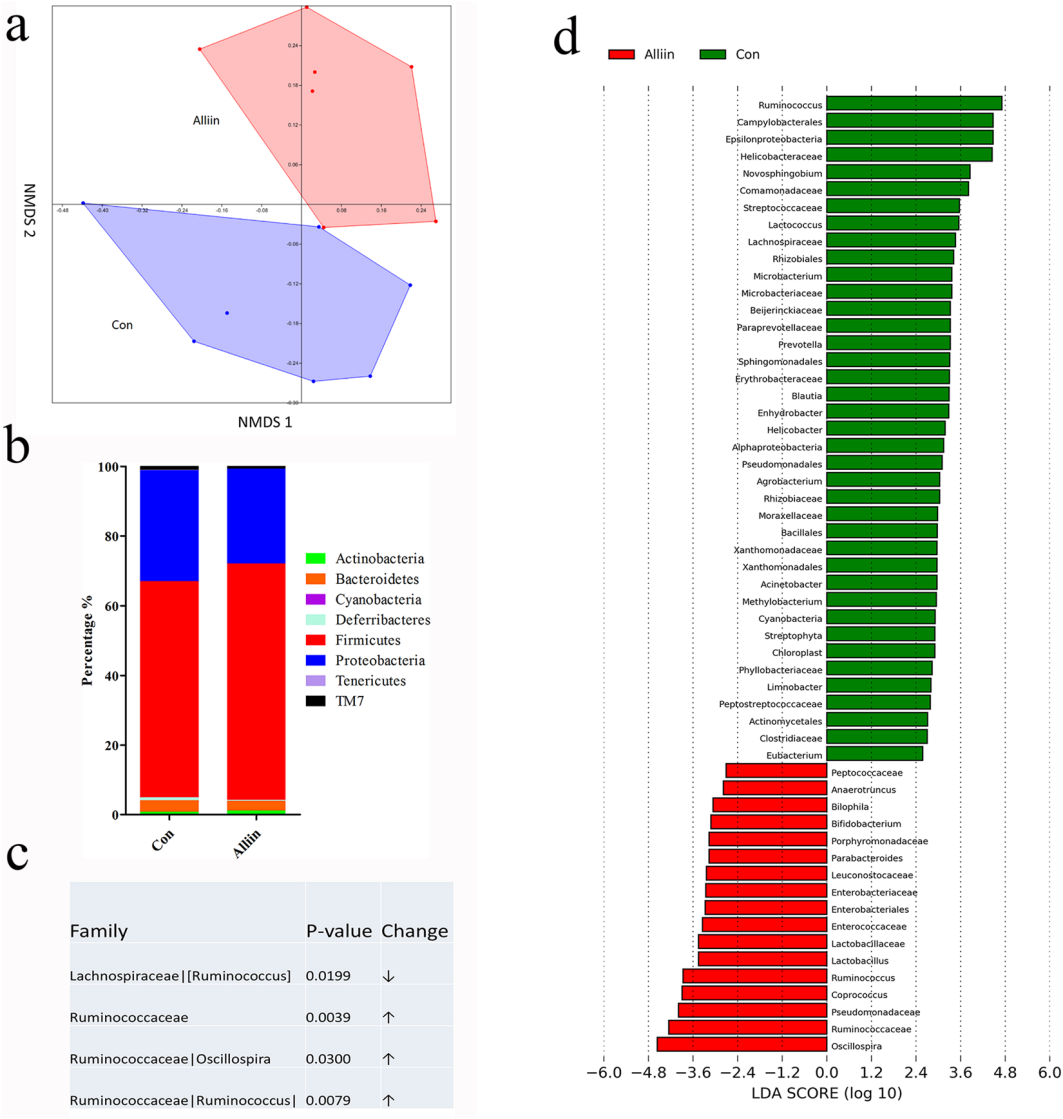
Analysis of intestinal microbiota. (**a**) NMD plots showed a significant difference in intestinal taxonomic composition between the alliin treated mice and the control. (**b**) In general, alliin increased the dominant bacteria of Actinobacteria and Firmicutes but decreased Bacteroidetes and Proteobacteria (no statistical difference). (**c**) Four groups of bacteria with significant differences were selected as in a table. (averaged reads over 100 and the percentage in its phylum over 1%). (**d**) Detailed abundance change was shown here. Red indicated that this taxon was more prevalent in the alliin group, while green indicated the taxon was more prevalent in the control group. Data are means ± SEM (n = 7).

Alliin modified the intestinal microbiota, which was significantly different between the alliin treated mice and the control (ANOSIM = 0.0008) (Fig. 5a). Specifically, alliin increased the dominant bacteria of *Actinobacteria* and *Firmicutes* but decreased *Bacteroidetes* and *Proteobacteria* at phylum, although the deference failed to reach the significant level (Fig. 5b). We selected two groups of bacteria with significant changes, of which the averaged reads were over 100 and their percentage was over 1% in their annotated phylum (Fig. 5c). The other changes with all annotated bacteria were plotted in Fig. 5d.

## Discussion

The beneficial effect of garlic or derived products has been widely reported, especially for those garlic commercial products publicized with garlicin. As a result, many suggest chop or crush garlic before consumption which can maximize the conversion of alliin into allicin. Indeed there are sufficient evidence showing the positive effect of allicin on metabolic disorders^7,9,19–22^. In fact the main bioactive ingredient of both fresh or dried garlic is alliin, the precursor of allicin^3^. Many medicinal effect of garlic products are probably, or at least partly, attributable to alliin, whereas the extent that alliin contributes to the beneficial function of garlic is not quite clear. Systematic studying of the nutraceutical or medicinal effect of alliin is lacking. Hereby our research systematically investigated the effect of the garlic derived compound alliin on the metabolic factors including body weight, glucose metabolism, adiposity, energy balance and associated metabolic risk factors.

We failed to observe any significant effect of alliin on the body weight or energy balance in the DIO mice. However, alliin was still a strong bioactive compound as it effectively reduced fasting glucose level, increased insulin sensitivity and improved the lipid profile of the DIO mice. As these symptoms were the key features of metabolic diseases, our result suggested that alliin had optimistic potential in nutraceutical or medicinal use. This was consistent with the previous report that alliin was comparatively effective as the anti-diabetic drugs such as glibenclamide and glyclazide^10,11^.

As reported we found alliin had a remarkable lipid lowing effect since it significantly decreased serum triglycerides and free fatty acids^13,23,24^. The different thing was the animal model we used were obese mice generated with high fat diet that imitated the food so readily available in society. Additionally, we found alliin increased serum HDL which is often referred as a type of “good” cholesterol as they can remove fat molecules. Previous reports showed that administration of garlic derived alliin at the dose of 200 mg/kg or 500 mg/kg body weight significantly decreased serum lipids and serum enzymes like liver glucose-6-phosphatase, intestinal HMG CoA reductase and liver hexokinase in addition to its hypoglycemic effect^23,25^. We obtained the similar effect in mice with free access to water containing a low concentration of alliin, under which condition the gavage associated stress was avoided as weight gain was thought to be facilitated by increased glucocorticoids^26^.

To ensure that the beneficial function of alliin was not caused by any toxic effect, we examined the organs as well as several serum biochemical factors. No apparent pathological change was observed in any organ examined in the DIO mice. Interestingly, we noticed that alliin might have the ability to protect against the liver damage evidenced by that the serum level of several markers like AST, ALP, TP and ALB were decreased within normal range by alliin treatment, which phenomenon was also found in alliin treated diabetic rats^11,25^.

Alliin contributed its hypoglycemic effect with no clear mechanism. Its ability of inhibiting glycolysis^23^, or stimulating insulin secretion^10^ may contribute to its hypoglycemic effect, while the nitric oxide was also involved in its regulation of glucose and lipid^27^. Although diallyl disulfide is one of the metabolites of allicin, it is not able of managing glucose metabolism alone^28^.

Another factor that is closely linked with metabolic diseases is the intestinal microbiota structure^29–31^. To examine this possible mechanism, we further analyzed the microbiota of the alliin treated DIO mice. We found several changes in intestinal bacterial composition after alliin treatment. Typically, alliin decreased *Lachnospiraceae* and increased *Ruminococcaceae*. Both *Lachnospiraceae* and *Ruminococcaceae* are the main gut bacteria family of mammal microbiota that have a high number of genes equipped to degrade a wide variety of polysaccharides including cellulose and hemicellulose components^32^. *Lachnospiraceae* is closely associated with insulin signaling in response to nutrient availability via the mammalian target of rapamycin (mTOR) and contributes to the development of diabetes^33^. This indicated that alliin regulated the glucose metabolism possibly via reducing the composition of *Lachnospiraceae* in the gut. On the other hand, the evidence associating *Ruminococcaceae* with metabolic disease is lacking. *Ruminococcaceae* is maybe involved in lipid metabolism as the high fat diet can decrease this family of bacteria^34^. Alliin that tended to increase *Ruminococcaceae* seemed to inhibit the negative effect caused by high fat foods. More evidence is still required to better understand the role of *Ruminococcaceae* in metabolic risks.

By systematically studying the metabolic factors in DIO mice model, we confirmed the hypoglycemic and hypolipidemic effect of alliin, the main bioactive compound in garlic. The beneficial function was at least partly attributed by modulation of the intestinal microbiota structure. As a relatively stable bioactive compound, alliin has high potential in use as nutraceutical and medicinal agents for hyperglycemia and hyperlipidemia.

## Methods

### Animals

Eight-week-old male C57BL/6 J mice (N = 20) from Vital River Laboratory Animal Technology (Beijing, China) were fed a high-fat (HFD, 60 kcal% fat) diet for 8 weeks. Water soluble alliin (0.1 mg/ml) was added to the drinking water. The mice were given either the alliin containing water or drinking water only for another 8 weeks. The mice were euthanized under mild ether anesthesia and their organs (liver, brown adipose tissue and SUB) were collected for further analysis. All animal studies were approved by the Animal Care and Use Committee in China Agricultural University and all experiments were performed in accordance with relevant guidelines and regulations.

### Glucose tolerance test and insulin tolerance test

Glucose tolerance test (GTT) and insulin tolerance test (ITT) were conducted at the 6th week. GTT procedure was reported previously^35^. ITT was carried out as follows: Mice were fasted for 4 h (9:00 A.M. to 1:00 P.M.) and insulin (1 U/kg Humulin R; Novo Nordisk) was administered intraperitoneally. The blood glucose levels were measured immediately before and after 15, 30, 45, and 60 min of insulin injection with an Accu-Chek glucose monitor (Roche Diagnostics, Indianapolis, IN, USA).

### Histological examination and hematoxylin-eosin staining

Tissues fixed with 4% paraformaldehyde were sectioned after embedded in paraffin. Multiple sections were prepared for hematoxylin-eosin staining, photographed (×20) and analyzed by a light microscope (DS-RI1; Nikon).

### Biochemical analysis of blood sample

Total cholesterol (CHO), triglyceride (TG), high density lipoprotein (HDL), low density lipoprotein (LDL), and free fatty acids (FFA) were determined using an RA-1000 autoanalyzer (Technicon, Tarrytown, NY, USA).

### Metabolic rate and physical activity

Respiratory exchange rate (RER) and physical activity were determined as previously described^35^. Briefly, the mice (n = 6) were acclimated to the TSE labmaster for approximately 24 h with dietary intake and drinks recorded, and then Vo_2_ and Vco_2_ were measured during the next 24 h. Voluntary activity of each mouse was measured with an optical beam technique (Opto-M3, Columbus Instruments, Columbus, OH, USA) over 24 h. Digested energy was analyzed as described previously^36^.

### Body composition measurement

The total fat mass, lean mass of free water of mice after 7 weeks treatment with high fat diet co-administered with either vehicle or alliin were assessed with the Small Animal Body Composition Analysis and Imaging System (MesoQMR23-060H-I; Nuimag Corp., Shanghai, China), according to the manufacturer’s instructions.

### Cold-induced thermogenesis

A cold tolerance test was performed in 13-week-old mice. The mice were placed in a cold chamber (4 °C) for up to 4 h with free access to food and water. Whole body temperature was monitored using infrared thermography, while core body temperature was measured with a rectal probe connected to a digital thermometer (Yellow Spring Instruments, Yellow Springs, OH, USA).

### Postitron emission-computed tomographic imaging

Positron emission-computed tomographic (PET-CT) imaging was achieved with the Siemens Inveon Dedicated PET (dPET) System and Inveon Multimodality (MM) System (CT-SPECT) (Siemens Preclinical Solutions, Knoxville, TN, USA). The detailed procedure was described previously^35^.

### Intestinal microflora analysis

Feces from mice were collected after alliin treatment and stored at −80 °C until use. Seven samples from each group were used for the intestinal microbiota analysis. Microbial genomic DNA was extracted from each fecal sample (0.1 g) using the method as previously described^37^. The V3 + V4 region of the 16S rRNA was amplified by PCR and sequenced using a HiSeq platform (Illumina, San Diego, CA, USA) at Novogene Bioinformatics Institute (Beijing, China). Sequence analyses were performed using Uparse software (Uparse v7.0.1001)^38^. Sequences with ≥97% similarity were assigned to the same operational taxonomic unit (OTU). Taxonomic annotation was conducted using a RDP classifier (Version 2.2)^39^. Nonmetric multidimensional scaling (NMDS) plots and ANOSIM were applied in analyzing the variation between each group utilizing the PAST version 2.17 software program. Community structure variance analysis was conducted with LEfSe software using the default parameters (http://huttenhower.sph.harvard.edu/galaxy/)^40^.

### Statistical analysis

Student t test was used to evaluate the differences between groups using IBM SPSS Statistics 20.0 software (IBM SPSS Inc., USA). Significant differences were defined as p < 0.05.

### Data availability

The datasets generated during and/or analysed during the current study are available from the corresponding author on reasonable request.
